# Response to growth hormone therapy in children with Noonan syndrome: correlation with or without *PTPN11* Gene mutation

**DOI:** 10.1186/1687-9856-2013-S1-P51

**Published:** 2013-10-03

**Authors:** Yoo-Mi Kim, Jin-Ho Choi, Beom Hee Lee, Chang-Woo Jung, Hye Young Jin, Jae-Min Kim, Gu-Hwan Kim, Jin Soon Hwang, Sei Won Yang, Jin Lee, Han-Wook Yoo

**Affiliations:** 1 Department of Pediatrics, Asan Medical Center Children’s Hospital, University of Ulsan College of Medicine, Seoul, Korea; 2 Medical Genetics Center, Asan Medical Center Children’s Hospital, University of Ulsan College of Medicine, Seoul, Korea; 3 Department of Pediatrics, Ajou University School of Medicine, Ajou University Hospital, Suwon, Korea; 4 Department of Pediatrics, Seoul National University College of Medicine, Seoul, Korea

## Aims

Noonan syndrome (NS) (MIM 163950) is an autosomal dominant disorder characterized by postnatal short stature, congenital heart disease, early feeding difficulties, mild learning disabilities, and characteristic facial dysmorphisms, short and webbed neck, and chest deformities. Proportionate short stature is well recognized as one of the key features of NS and has been reported in more than 80% of patients affected by NS. The objective of this study was to evaluate the efficacy of recombinant human growth hormone (rhGH) therapy and the influence of genotype on the response to rhGH therapy in children with Noonan syndrome (NS).

## Methods

Fourteen male and four female subjects with NS with short stature whose height was less than third percentile, were included. The rhGH was subcutaneously administered at a dose of 66 μg/kg/day, subcutaneously for a 12-month period. Mutations in the *PTPN11* gene were identified in 10 subjects (55.6%). Mutations in the *SOS1* (two children, 11.1%), *MEK1* (one child, 5.6%) and *KRAS* (one child, 5.6%) genes were also found.

## Results

The mean age was 8.3±2.4 years (range, 4.4 to 13.2 years) at the start of rhGH treatment. Height-SDS increased from -2.8±0.9 at the start of rhGH therapy to -2.0±0.9 12 months later (*P*<0.001). Height velocity increased from 5.0±0.9 cm/year in the year before treatment to 8.9±1.6 during treatment (*P*<0.001). Changes in height SDS, height velocity, and serum IGF-1 level did not differ significantly between those children with or without *PTPN11* mutations.

## Conclusion

The rhGH therapy significantly improved the growth velocity and increased the serum IGF-1 level. Long-term correlation between genotype and rhGH therapy responsiveness needs to be addressed in a large population.

